# Lack of Association of Coffee Consumption with the Prevalence of Self-Reported Type 2 Diabetes Mellitus in a Mexican Population: A Cross-Sectional Study

**DOI:** 10.3390/ijerph15102100

**Published:** 2018-09-25

**Authors:** Ana Karen Gil-Madrigal, Thelma Beatriz González-Castro, Carlos Alfonso Tovilla-Zárate, Daniela Georgina Aguilar-Velázquez, Tania Guadalupe Gómez-Peralta, Isela Esther Juárez-Rojop, María Lilia López-Narváez, Elizabeth Carmona-Díaz, Ana Fresan, Jorge Luis Ble-Castillo, Antonia Pérez-Mandujano, Crystell Guzman-Priego

**Affiliations:** 1 División Académica Multidisciplinaria de Comalcalco, Universidad Juárez Autónoma de Tabasco, Comalcalco 86650, Tabasco, Mexico, karen_gmadrigal@hotmail.com (A.K.G.-M.); daniela-azul@hotmail.com (D.G.A.-V.); tanyperalta93@gmail.com (T.G.G.-P.); elizadiaz1@hotmail.com (E.C.-D.); 2 División Académica Multidisciplinaria de Jalpa de Méndez, Universidad Juárez Autónoma de Tabasco, Jalpa de Méndez 86205, Tabasco, Mexico; thelma.glez.castro@gmail.com; 3 División Académica de Ciencias de la Salud, Universidad Juárez Autónoma de Tabasco, Villahermosa 86100, Tabasco, Mexico; jblecastillo@hotmail.com (J.L.B.-C.); antony-jano@hotmail.com (A.P.-M.); crystell_guzman@hotmail.com (C.G.-P.); 4 Hospital General de Yajalón, Secretaría de Salud, Yajalón 29930, Chiapas, Mexico; dralilialonar@yahoo.com.mx; 5 Subdirección de Investigaciones Clínicas, Instituto Nacional de Psiquiatría Ramón de la Fuente Muñiz, Ciudad de México 14370, Mexico; fresan@imp.edu.mx

**Keywords:** diabetes, coffee, Mexican population

## Abstract

It is estimated that almost 366 million people are currently suffering from diabetes mellitus worldwide. However, it has been suggested that coffee consumption has a protective effect against the development of type 2 diabetes mellitus. This association has been observed in many regions around the world. Today, there are no reports in Mexico regarding this association. Therefore, the aim of this study was to assess the association between coffee intake and self-reported type 2 diabetes mellitus in the southeastern part of Mexico. This study included 1277 residents of Comalcalco, a municipality of Tabasco State, Mexico. We calculated the prevalence for diabetes and performed multivariate analysis using multiple logistic regressions to evaluate the combined association with type 2 diabetes mellitus. The prevalence of the diabetes was 12.52% (95% CI: 10.67–14.38). The majority of people surveyed (77.29%; 95% CI: 74.95–79.60) indicated they were coffee drinkers. The results of multivariate analysis showed a non-significant relationship between the number of cups of coffee drank and type 2 diabetes mellitus. The adjusted odds ratio gave the following values: 1.20, (95% CI: 0.59–2.41) for non-daily consumption; 1.66 (0.82–3.34), for 1 cup of coffee peer day, and 1.49 (0.78–2.86) for 2–3 cups. Subsequently, an adjustment was made for age, gender, marital status, education, alcohol consumption, and cigarette smoking. In our population, we did not observe an association between coffee intake and its protective relationship with self-reported type 2 diabetes mellitus.

## 1. Introduction

The global prevalence of diabetes mellitus is continuously rising. Nowadays, almost 366 million people worldwide have diabetes mellitus; it is estimated that by 2030 diabetes mellitus will affect 552 million people [1]. In Mexico, the prevalence of this disease is on the rise. In 1993, the prevalence of diabetes mellitus was 4.6%; in 2007, it increased to 7.30%; in 2012, 9.17% of the general population had the disease [2,3,4]. 

Coffee is the most consumed beverage in the world. It is a complex mixture of thousands of compounds including caffeine, phenolic compounds, niacin, minerals, and fiber [5]. With regard to diabetes, the literature suggests that sub-clinical inflammation has been implicated in the development of insulin resistance and coffee consumption may improve insulin sensitivity by decreasing inflammation [6,7,8] (for a more detailed description of the mechanism, see [5,9]). Recently, many studies have encountered a protective association between coffee consumption and type 2 diabetes mellitus [6,10,11,12,13,14]. This protective effect has been observed in different countries and populations such as African American women [10], populations of California, Florida, Louisiana, New Jersey, North Carolina, and Pennsylvania [15], and other states of the American Union [12], as well as the Taiwan-Chinese population [11], the Brazilian population [16], the Singapore-Chinese population [17], the Malay-Chinese and Indian populations [6], and other population samples [18,19,20]. Moreover, a first meta-analysis reported that habitual coffee consumption was associated with a substantial lower risk for type 2 diabetes [21]. Similarly, in a second meta-analysis, high intake of coffee was associated with a reduced risk for diabetes [22,23]. However, at the present time, there are no reports in the Mexican population evaluating the association between coffee consumption and diabetes mellitus. The consumption of coffee per capita in Mexico is 1.41 kg every year [24]. This consumption is much lower than that reported for developing countries [25]. Additionally, Mexico is among the countries with major prevalence of diabetes mellitus [26]. Thus, it is important know the association between coffee consumption and prevalence of self-reported type 2 diabetes in the Mexican population, particularly when designing programs to prevent or treat diabetes mellitus in the Mexican population. We performed a cross-sectional association study to assess the possible protective effect of coffee intake against type 2 diabetes mellitus in a population of Southeastern Mexico who self-reported having diabetes. 

## 2. Materials and Methods

### 2.1. Participants

We designed a cross-sectional population-based survey for individuals living in Comalcalco. Comalcalco is a municipality of Tabasco with 41,458 inhabitants, which represented 21.5% of the overall population of Tabasco State at the moment of the study. The inclusion criteria were individuals 18–79 years of age and residents of the Comalcalco municipality. The exclusion criteria were individuals who did not complete the interview, and individuals who did not sign the informed consent. In a simple random sampling, a total of 1400 people were surveyed. However, only 1277 people completed the inclusion criteria and were enrolled in this study. 

### 2.2. Ethics Statement

We only surveyed individuals descending from Mexican parents. The retrieved data were confidential. Before the interview, participants were informed about the aims of the study and signed a declaration of informed consent. This study was conducted according to the guidelines laid down in the Declaration of Helsinki. This study was approved by the DAMC-UJAT Ethics and Research Committee (UJAT-DAMC-2012-03), as well as by the ethics committee of the Ministry of Health of Tabasco (INV/262/C/0512). 

### 2.3. Data Collection

We designed a cross-sectional population-based survey and a questionnaire to obtain information regarding different habits and health conditions between healthy individuals and people with type 2 diabetes. A semi-structured questionnaire was designed specifically for this study, which was used to collect detailed data of sociodemographic characteristic (gender, age, socio-behavioral aspects, years of schooling), health habits (cigarette smoking and alcohol intake), and coffee consumption (Cronbach’s alpha 0.69). The frequency of coffee intake ranged from none to six cups or more per day. However, the number of participants who drank more than 5 cups per day was small; therefore; we decided to change the category of 4–6 cups per day into ≥4 cups per day (4 cups, *n* = 9, 5 cups, *n* = 36, 6 cups, *n* = 7). For the analysis, we combined two types of coffee consumption: instant and filtered. Participants were interviewed by nurses and medical students. The questionnaire was applied in person. For sample calculation, we used an a priori model, α = 0.05, and 70% prevalence for coffee intake was considered. Precision of 2.0% was adopted. Sample size = 1188, with a power of 0.95.

### 2.4. Anthropometric Measurements

The following parameters were collected: height, weight, and blood pressure. These measurements were collected as previously reported [27].

### 2.5. Definition of Type 2 Diabetes 

Self-reported diagnoses of diabetes were rendered by the participants. During the assessment of self-reported diagnosis, the time of onset of diabetes (in years) was obtained. In addition, a supplementary questionnaire regarding symptoms was applied to confirm the diagnosis of this illness. Glucose levels in plasma were not measured. 

### 2.6. Definition of Use of Substances

History of alcohol consumption per week was asked. The person with regular weekend use or sporadic use of alcoholic beverages was considered an alcohol consumer. Individuals who smoke at least 10 cigarettes per week were classified as smokers. Carbon monoxide in breath was not measured. People consuming alcoholic beverages or cigarettes were considered users of substances.

### 2.7. Statistical Analysis

Descriptive statistics was used to characterize the sample using frequencies and percentages for categorical variables and mean ± standard deviations for continuous variables. The Kolmogorov–Smirnov test was used to analyze whether the continuous variables were under normal distribution. The Kolmogorov–Smirnov test showed *p*-values ≥ 0.20). Dummy codification was used for continuous variables to perform comparative analyses using chi-square tests and obtain estimated odd ratios (95% CI) for individual variables in association with diabetes. Age was categorized using a cutoff value of up to 50 as previously reported [27]. For level of education, six years of elementary school was the value to categorize the sample as this level of education is considered the minimum to have in Mexico. BMI was dichotomized in normal and overweight/obesity in accordance with international obesity task force criteria, and systolic and diastolic BP values were categorized according to Mexican official norm NOM-030-SSA2-1999. Subsequently, multivariate analysis using multiple logistic regressions was performed to evaluate the combined relationship of several factors associated with diabetes among the overall population after adjusting for confounding variables (in this analysis we included socio-demographic, anthropometric, and clinic characteristics as explanatory variables). Results are presented as adjusted odds ratios (AOR) with 95% CI, which express the magnitude of the relationship of each category on the outcome relative to the reference category. Prevalence was calculated using EPIDAT 3.1 software (software for epidemiologic analysis of tabulated data, v. 3.1). Other calculations were performed with the SPSS version 14.0 software (SPSS Inc., Chicago, IL, USA). The level of significance was set at *p* = 0.05. 

## 3. Results 

### 3.1. Descriptive Characteristics

The socio-demographic characteristics of the participants are summarized in Table 1. The larger number of participants were females (53.20%); of these, 54.0% were married and 36.20% were single. The main occupations were classified as part-time job (41.00%) and housewife (29.00%). The mean age was 37.27 ± 13.44 years old with a range of 18–79. The mean level of education was 5.01 ± 4.63 years, with a range of 0–21 years of school education. The mean body mass index was 27.92 ± 4.31 (range 20–45.87). The mean systolic BP was 116.28 ± 11.12 (range 70–170) and the mean diastolic BP was 78.22 ± 9.78 (Table 1). Significant differences emerged between groups in terms marital status, occupation, age, education, and body mass index (Table 1). Patients with diabetes mellitus were predominantly married. They worked full time and had a higher body mass index. 

### 3.2. Prevalence of Type 2 Diabetes and Obesity

In the overall population, 12.52% (95% CI: 10.67–14.38) were positive for diabetes. The prevalence of type 2 diabetes was 15.52% (95% CI 12.26–18.80) in males and 12.90% (95% CI: 10.17–15.64) in females.

### 3.3. Coffee Consumption

In our sample, from the 1277 participants, 77.29% (95% CI: 74.95–79.60) were coffee drinkers. 

Table 2 summarizes the results of univariate and multivariate analyses for the relationship between the number of cups of coffee per day and the presence of diabetes. No relation between coffee consumption and type 2 diabetes was observed. When compared with the daily consumption of 4 or more cups of coffee, we found the following adjusted odd ratio (AOR) values: 1.20 (95% CI 0.59–2.41) for non-daily consumption, 1.66 (0.82–3.34) for 1 cup daily consumption, and 1.49 (0.78–2.86) for to 2–3 cups. 

## 4. Discussion

From 2000 to 2007, 477,036 individuals died of diabetes mellitus in Mexico [28]. Diabetes mellitus was the second cause of death claiming 9.7% of total deaths [29]. The mortality rate increased from 77.9 in 2000 to 89.2 in 2007 per 100,000 inhabitants [28]. In consequence, we estimated the prevalence of type 2 diabetes mellitus in our population and analyzed the prevalence of coffee consumption for a possible association between coffee intake and a Tabascan population who self-reported type 2 diabetes mellitus. To our knowledge, this is the first study evaluating this association in the Mexican population. The present study failed to detect any association between coffee consumption and a decrease of self-reported type 2 diabetes mellitus.

In the population studied, we found a diabetes mellitus prevalence of 12.52%. Although it is considered high, it was an expected result, because the National Survey of Healthy and Nutrition 2012 showed that the prevalence of diabetes mellitus in Tabasco State is higher (9.4%) than the national average (9.2%) [30]. Many factors could be associated to this high prevalence i.e., the diet of the local population [31]. In recent years, coffee intake has been associated with a lower risk for type 2 diabetes in several studies [12,19,32,33,34]. This has been observed in caffeinated and decaffeinated coffee [35]. However, in our study, we did not encounter evidence for an association between coffee consumption and self-reported type 2 diabetes. There is only one other study that has reported this lack of association [36]. It is likely that the geographic localization of the sample where the study was performed and the tribal customs related to coffee consumption may have contributed to this lack of association [37]. 

There are a number of possible reasons for the lack of association between coffee consumption and the protective association with type 2 diabetes mellitus that we considered. First, the number of participants in our study may not be large enough to show a difference between the coffee consumption and the presence of self-reported type 2 diabetes. Second, alcohol and cigarette smoking were a covariate that influenced our results, since the prevalence of substance use in our sample was 31%, which is higher than what has been reported in other Mexican populations [38]. Other possible explanation may be that the inverse association between coffee consumption and diabetes was observed only in a specific age group. For example, in our study, the mean age was 37.27 ± 13.44 years old, and studies in other populations included older patients: 50–70 years of age [15], 40–60 years old [11,39], and 45–75 years of age [34,37]. Another possibility may be the small consumption of coffee in our population. The average coffee consumption in the Mexican population is only 161 mL per day in individuals 19 years old and older [40,41]. This amount is lower than the standard coffee cup serving size of 240 mL. However, the inverse association between coffee consumption and diabetes has been observed in China, which is a country with a coffee consumption rate similar to that of Mexico [11]. Interestingly, a non-protective effect of coffee consumption in type 2 diabetes has been observed in studies that evaluated the association between coffee consumption and coronary heart disease in a Mexican population [42].

As a limitation of our study, glucose levels were not quantified to confirm the diagnosis of diabetes mellitus. Second, we did not utilize a questionnaire to evaluate food intake and physical activity. Third, in North America and the Caribbean, it has been estimated that 29.2% of the population have been undiagnosed with diabetes mellitus [1], and this can lead to outcome misclassification and attenuation of risk estimates. Fourth, people who drank decaffeinated coffee were not evaluated. Future studies are needed to explore the role of decaffeinated coffee and type 2 diabetes mellitus in our population. Fifth, the long-term relationship between coffee consumption and type 2 diabetes mellitus prevention in older individuals was not evaluated. Our results should not be generalized for the entire Mexican population, as only individuals of one city were evaluated. The most important limitation of the present study is its cross-sectional design. Even though our results showed no association between coffee intake and diabetes, future longitudinal studies, specifically a cohort study, may give clearer results about this lack of association or even show that there can be an association that is evidenced with the passage of time.

## 5. Conclusions

In conclusion, our results yielded a lack of association between coffee intake and self-reported type 2 diabetes in a population of Southeastern Mexico. However, low coffee consumption in our population, sample size, average age, and methodological differences with other studies contributed to the lack of association, and future studies that overcome these limitations should be carried out. 

## Figures and Tables

**Table 1:** Association between socio-demographic and clinical characteristics divided by type 2 diabetes of the populations in the study.

Characteristics		All Sample *n* (%)	With Diabetes	Without Diabetes	OR (95% CI)	*p*-Value
Gender	Male	597 (46.80)	69 (43.10)	528 (47.30)	0.84 (0.60–1.18)	0.32
	Female	680 (53.20)	91 (56.90)	589 (52.70)		
Marital Status	Married	690 (54.00)	100 (62.50)	590 (52.80)		**<0.01**
	Single	463 (36.20)	37 (23.10)	426 (38.10)		
	Widowed	67 (5.20)	10 (6.30)	57 (5.10)		
	Separated/divorced	57 (4.50)	13 (8.10)	44 (3.90)		
Occupation	Unemployed	38 (3.00)	5 (3.10)	33 (3.00)		**<0.001**
	Housewife	370 (29.00)	47 (29.70)	323 (28.90)		
	Student	70 (5.50)	3 (1.90)	67 (6.00)		
	Half-time job	524 (41.00)	29 (18.10)	495 (44.30)		
	Full-time job	275 (21.50)	76 (47.50)	199 (17.80)		
Age (in years)	Up to 49	1051 (82.30)	99 (61.90)	952 (85.20)	**0.28 (0.19–0.40)**	**<0.001**
	>50	226 (17.70)	61 (38.10)	165 (14.80)		
Education	Up to 6 years of schooling	807 (63.20)	84 (52.50)	723 (64.70)	**0.60 (0.43–0.84)**	**<0.01**
	>6 years of schooling	470 (36.80)	76 (47.50)	394 (35.30)		
Body mass index	Up to 25	460 (36.00)	38 (23.80)	422 (37.80)	**0.51 (0.34–0.75)**	**<0.001**
	>25	817 (64.00)	122 (76.30)	695 (62.20)		
Use of substances	Yes	397 (31.10)	59 (36.90)	338 (30.30)	1.34 (0.95–1.90)	0.09
	No	880 (68.90)	101 (63.10)	779 (69.70)		
Systolic BP (mmHg)	Up to 120	1073 (84.00)	126 (78.80)	947 (84.80)	0.66 (0.44–1.00)	0.05
	>120	204 (16.00)	34 (21.30)	170 (15.20)		
Diastolic BP (mmHg)	Up to 80 mmHg	1024 (80.20)	123 (76.90)	901 (80.70)	0.79 (0.53–1.18)	0.26
	>80 mmHg	253 (19.8)	37 (23.1)	216 (19.3)		

significance in bold.

**Table 2:** Relationship between coffee consumption and prevalence of diabetes after adjustment for potential confounding factors.

Coffee Consumption	No. of People *n* = 1277	Prevalence of Diabetes *n* (%)	Without Diabetes *n* (%)	OR (95% CI)	Multivariate OR (95% CI), *p* Value *
Nondaily	290 (22.70)	36 (12.40)	254 (87.60)	1.31 (0.67–2.57)	1.20 (0.59–2.41), 0.60
1 cup/d	248 (19.40)	38 (15.30)	210 (84.70)	1.68 (0.86–3.28)	1.66 (0.82–3.34), 0.15
2–3 cups/d	605 (47.40)	73 (12.10)	532 (87.90)	1.27 (0.68–2.37)	1.49 (0.78–2.86), 0.22
≥4 cups/d	134 (10.50)	13 (9.70)	121 (90.30)	Reference	Reference

* Adjusted OR for age, body mass index, gender, marital status, education, systolic BP, diastolic BP, alcohol consumption, and smoking habit.

## References

[1] Federation I.D. (2011). Idf Diabetes Atlas.

[2] Olaiz G., Rivera J., Shamah T., Rojas R., Villalpando S., Hernández M., Sepúlveda J. (2006). Encuesta Nacional de Salud y Nutrición 2006.

[3] Barquera S., Campos-Nonato I., Aguilar-Salinas C., Lopez-Ridaura R., Arredondo A., Rivera-Dommarco J. (2013). Diabetes in Mexico: Cost and management of diabetes and its complications and challenges for health policy. Glob. Health.

[4] Hernández-Ávila M., Gutiérrez J.P., Reynoso-Noverón N. (2013). Diabetes mellitus en México. El estado de la epidemia. Salud Publica de Mexico.

[5] Natella F., Scaccini C. (2012). Role of coffee in modulation of diabetes risk. Nutr. Rev..

[6] Rebello S.A., Chen C.H., Naidoo N., Xu W., Lee J., Chia K.S., Tai E.S., van Dam R.M. (2011). Coffee and tea consumption in relation to inflammation and basal glucose metabolism in a multi-ethnic Asian population: A cross-sectional study. Nutr. J..

[7] Kempf K., Herder C., Erlund I., Kolb H., Martin S., Carstensen M., Koenig W., Sundvall J., Bidel S., Kuha S. (2010). Effects of coffee consumption on subclinical inflammation and other risk factors for type 2 diabetes: A clinical trial. Am. J. Clin. Nutr..

[8] Wang H., Jeong H., Kim N.H., Kang Y., Hwang K., Lee H., Hong J.H., Oh K.S. (2018). Association between beverage intake and obesity in children: The Korea National Health and Nutrition Examination Survey (KNHANES) 2013–2015. Nutr. Res. Pract..

[9] Pimentel G.D., Zemdegs J.C., Theodoro J.A., Mota J.F. (2009). Does long-term coffee intake reduce type 2 diabetes mellitus risk?. Diabetol. Metab. Syndr..

[10] Boggs D.A., Rosenberg L., Ruiz-Narvaez E.A., Palmer J.R. (2010). Coffee, tea, and alcohol intake in relation to risk of type 2 diabetes in African American women. Am. J. Clin. Nutr..

[11] Lin W.Y., Xaiver Pi-Sunyer F., Chen C.C., Davidson L.E., Liu C.S., Li T.C., Wu M.F., Li C.I., Chen W., Lin C.C. (2011). Coffee consumption is inversely associated with type 2 diabetes in Chinese. Eur. J. Clin. Investig..

[12] Bhupathiraju S.N., Pan A., Malik V.S., Manson J.E., Willett W.C., van Dam R.M., Hu F.B. (2013). Caffeinated and caffeine-free beverages and risk of type 2 diabetes. Am. J. Clin. Nutr..

[13] Ohnaka K., Ikeda M., Maki T., Okada T., Shimazoe T., Adachi M., Nomura M., Takayanagi R., Kono S. (2012). Effects of 16-week consumption of caffeinated and decaffeinated instant coffee on glucose metabolism in a randomized controlled trial. J. Nutr. Metab..

[14] Ericson U., Hindy G., Drake I., Schulz C.A., Brunkwall L., Hellstrand S., Almgren P., Orho-Melander M. (2018). Dietary and genetic risk scores and incidence of type 2 diabetes. Genes Nutr..

[15] Freedman N.D., Park Y., Abnet C.C., Hollenbeck A.R., Sinha R. (2012). Association of coffee drinking with total and cause-specific mortality. N. Engl. J. Med..

[16] Machado L.M., da Costa T.H., da Silva E.F., Dorea J.G. (2011). Association of moderate coffee intake with self-reported diabetes among urban Brazilians. Int. J. Environ. Res. Public Health.

[17] Odegaard A.O., Pereira M.A., Koh W.P., Arakawa K., Lee H.P., Yu M.C. (2008). Coffee, tea, and incident type 2 diabetes: The singapore chinese health study. Am. J. Clin. Nutr..

[18] Pereira M.A., Parker E.D., Folsom A.R. (2006). Coffee consumption and risk of type 2 diabetes mellitus: An 11-year prospective study of 28,812 postmenopausal women. Arch. Intern. Med..

[19] Van Dam R.M., Feskens E.J. (2002). Coffee consumption and risk of type 2 diabetes mellitus. Lancet.

[20] Torres-Collado L., García-de la Hera M., Navarrete-Muñoz E., Compañ-Gabucio L., Gonzalez-Palacios S., Vioque J. (2018). Coffee drinking and associated factors in an elderly population in Spain. Int. J. Environ. Res. Public Health.

[21] van Dam R.M., Hu F.B. (2005). Coffee consumption and risk of type 2 diabetes: A systematic review. JAMA.

[22] Huxley R., Lee C.M., Barzi F., Timmermeister L., Czernichow S., Perkovic V., Grobbee D.E., Batty D., Woodward M. (2009). Coffee, decaffeinated coffee, and tea consumption in relation to incident type 2 diabetes mellitus: A systematic review with meta-analysis. Arch. Intern. Med..

[23] Carlstrom M., Larsson S.C. (2018). Coffee consumption and reduced risk of developing type 2 diabetes: A systematic review with meta-analysis. Nutr. Rev..

[24] Aceves-Navarro L.A., Rivera-Hernández B., López-Castañeda A., Palma-López D.J., González-Mancillas R., Juárez-López J.F. (2018). Potential areas and vulnerability of the robust coffee crop (*Coffea canephora* P.) to climate change in the state of tabasco, Mexico. Nova Sci..

[25] Chamorro A.C.A., Benavides N.A.B. (2018). El mercado del café en los contextos mundial, nacional y regional. Rev. UNIMAR.

[26] Rojas-Martínez R., Basto-Abreu A., Aguilar-Salinas C.A., Zárate-Rojas E., Villalpando S., Barrientos-Gutiérrez T. (2018). Prevalence of previously diagnosed diabetes mellitus in Mexico. Salud Publica de Mexico.

[27] Tovilla-Zarate C., Juarez-Rojop I., Peralta Jimenez Y., Jimenez M.A., Vazquez S., Bermudez-Ocana D., Ramon-Frias T., Genis Mendoza A.D., Garcia S.P., Narvaez L.L. (2012). Prevalence of anxiety and depression among outpatients with type 2 diabetes in the Mexican population. PLoS ONE.

[28] Sanchez-Barriga J.J. (2010). Mortality trends from diabetes mellitus in the seven socioeconomic regions of Mexico, 2000–2007. Rev. Panam. Salud Publ..

[29] Stevens G., Dias R.H., Thomas K.J.A., Rivera J.A., Carvalho N., Barquera S., Hill K., Ezzati M. (2008). Characterizing the epidemiological transition in Mexico: National and subnational burden of diseases, injuries, and risk factors. PLoS Med..

[30] (2013). Encuesta Nacional de Salud y Nutrición 2012. Resultados Por Entidad Federativa, Tabasco.

[31] Navarrete-Cortes A., Ble-Castillo J.L., Guerrero-Romero F., Cordova-Uscanga R., Juarez-Rojop I.E., Aguilar-Mariscal H., Tovilla-Zarate C.A., Lopez-Guevara Mdel R. (2014). No effect of magnesium supplementation on metabolic control and insulin sensitivity in type 2 diabetic patients with normomagnesemia. Magnes. Res. Off. Organ Int. Soc. Dev. Res. Magnes..

[32] Salazar-Martinez E., Willett W.C., Ascherio A., Manson J.E., Leitzmann M.F., Stampfer M.J., Hu F.B. (2004). Coffee consumption and risk for type 2 diabetes mellitus. Ann. Intern. Med..

[33] van Dam R.M., Willett W.C., Manson J.E., Hu F.B. (2006). Coffee, caffeine, and risk of type 2 diabetes: A prospective cohort study in younger and middle-aged U.S. Women. Diabetes Care.

[34] Doo T., Morimoto Y., Steinbrecher A., Kolonel L.N., Maskarinec G. (2013). Coffee intake and risk of type 2 diabetes: The multiethnic cohort. Public Health Nutr..

[35] Ding M., Bhupathiraju S.N., Chen M., van Dam R.M., Hu F.B. (2014). Caffeinated and decaffeinated coffee consumption and risk of type 2 diabetes: A systematic review and a dose-response meta-analysis. Diabetes Care.

[36] Saremi A., Tulloch-Reid M., Knowler W.C. (2003). Coffee consumption and the incidence of type 2 diabetes. Diabetes Care.

[37] Zhang Y., Lee E.T., Cowan L.D., Fabsitz R.R., Howard B.V. (2011). Coffee consumption and the incidence of type 2 diabetes in men and women with normal glucose tolerance: The strong heart study. Nutr. Metab. Cardiovasc. Dis..

[38] Lee J.H., Oh M.K., Lim J.T., Kim H.G., Lee W.J. (2016). Effect of coffee consumption on the progression of type 2 diabetes mellitus among prediabetic individuals. Korean J. Fam. Med..

[39] Lin C.C., Liu C.S., Lai M.M., Li C.I., Chen C.C., Chang P.C., Lin W.Y., Lee Y.D., Lin T., Li T.C. (2007). Metabolic syndrome in a taiwanese metropolitan adult population. BMC Public Health.

[40] Current Worlwide Annual Coffee Consumption per Capita. http://chartsbin.com/view/581.

[41] Rivera J.A., Munoz-Hernandez O., Rosas-Peralta M., Aguilar-Salinas C.A., Popkin B.M., Willett W.C. (2008). Beverage consumption for a healthy life: Recommendations for the mexican population. Salud Publica de Mexico.

[42] MacKenzie G., Greig M., Hay I., Pemberton J. (2017). Competing risk analysis of factors related to long-term incidence of chd. J. Epidemiol. Commun. Health.

